# Development of a MALDI-TOF MS-based screening panel for accelerated differential detection of carbapenemases in *Enterobacterales* using the direct-on-target microdroplet growth assay

**DOI:** 10.1038/s41598-020-61890-7

**Published:** 2020-03-19

**Authors:** Carlos L. Correa-Martínez, Evgeny A. Idelevich, Katrin Sparbier, Thorsten Kuczius, Markus Kostrzewa, Karsten Becker

**Affiliations:** 1 Institute of Medical Microbiology, University Hospital Münster, Münster, Germany; 2 Institute of Hygiene, University Hospital Münster, Münster, Germany; 3 Bruker Daltonik GmbH, Bremen, Germany; 4 Friedrich Loeffler-Institute of Medical Microbiology, University Medicine Greifswald, Greifswald, Germany

**Keywords:** Microbiology techniques, Bacterial infection

## Abstract

Carbapenemase-producing bacteria are a growing issue worldwide. Most phenotypic detection methods are culture-based, requiring long incubation times. We present a phenotypic screening panel for detection of carbapenem non-susceptibility and differentiation of carbapenemase classes and AmpC, the MALDI-TOF MS-based direct-on-target microdroplet growth assay (DOT-MGA). It was validated on 7 reference strains and 20 challenge *Enterobacterales* isolates. Broth microdilution (BMD) and combination disk test (CDT) were also performed, as well as PCR as reference method. The panel based on the synergy between meropenem and carbapenemase inhibitors, determined by incubating these substances with bacterial suspension on a MALDI-TOF MS target and subsequently assessing bacterial growth on the target’s spots by MS. After 4 hours of incubation, DOT-MGA correctly identified KPC, MBL and OXA (100% agreement with PCR). Detection of AmpC coincided with BMD and CDT but agreement with PCR was low, not ruling out false negative PCR results. DOT-MGA delivered more accurate results than BMD and CDT in a significantly shorter time, allowing for detection of carbapenem non-susceptibility, MIC determination and carbapenemase differentiation in one step.

## Introduction

The time to availability of microbiological results plays a critical role in the medical decision-making process as it has a direct impact on the choice of antimicrobial therapy. Advanced antimicrobial susceptibility testing (AST) methods may help to accelerate the targeted antimicrobial therapy^1^. However, the implications of a rapid and accurate laboratory diagnosis go beyond the clinical aspects: the consumption of antimicrobial substances as well as the rate of the development of bacterial resistance have shown to be directly affected by the information provided by the microbiology laboratory^2^. An association has also been observed between aspects concerning hospitalisation (amount of resources invested, total length of stay) and the accuracy of the implemented therapeutic strategies, which in turn depends on the turnaround times of the diagnostic tools employed^3–6^. Most importantly, a prompt detection of resistance patterns leads to better clinical outcomes^3,7,8^.

Despite advances in phenotypic AST methodology, the time required for bacteria to replicate remained a natural obstacle, as reaching the stationary growth phase is required for the performance and interpretation of most available antibiotic susceptibility tests. This translates into incubation times exceeding 10 hours for culture-based diagnostic methods, a time lapse during which therapeutic decisions are taken empirically. Thus, developing new diagnostic methods that tackle the delay posed by the usual incubation times should be prioritised, especially in an era of increasing antimicrobial resistance^1^.

As resistance against antibiotics raises concern worldwide, carbapenem resistance stands out as one of the most preoccupying edges of this problem, seeing as how carbapenems constitute the most reliable treatment option for infections caused by multidrug-resistant (including ESBL-producing) Gram-negative bacteria, particularly *Enterobacteriaceae* and other *Enterobacterales*^9^. First described in the early 1990s^10^, resistance against carbapenems has reached a high incidence in various regions^9,11,12^.

Several rapid molecular, colourimetric and mass spectrometry-based methods have been developed and represent an alternative to the growth-based techniques, offering results in a shorter time. However, various drawbacks hampered their widespread use in the routine laboratory. Concerning PCR-based approaches, a limited number of targeted genes and the inability to detect emerging ones represent the main disadvantages of these genotypic techniques^13^. Colourimetric methods provide no information on the specific carbapenemase type present^13^ and have been reported to display a low sensitivity for the detection OXA-48 producers^14,15^.

The applicability of matrix-assisted laser desorption ionisation time-of-flight mass spectrometry (MALDI-TOF MS) for susceptibility testing has been demonstrated in recent years, based on: (i) monitoring the mass shifts that derived from antibiotic hydrolysis^16–18^; (ii) detecting the incorporation of isotopically labelled aminoacids by resistant bacteria growing in presence of carbapenems^19^; (iii) analysing the biomass present after cultivating bacteria in presence or absence of carbapenems^20^; or (iv) the recently described direct-on-target microdroplet growth assay (DOT-MGA)^21–23^.

Here, we propose a rapid all-in-one MALDI-TOF MS-supported screening panel based on the DOT-MGA. The panel allows to detect carbapenem non-susceptibility as well as to make a differential identification of carbapenemase classes. A validation of the method was carried out with 20 carbapenem-non-susceptible *Enterobacterales* strains.

## Results

**Figure 1:**
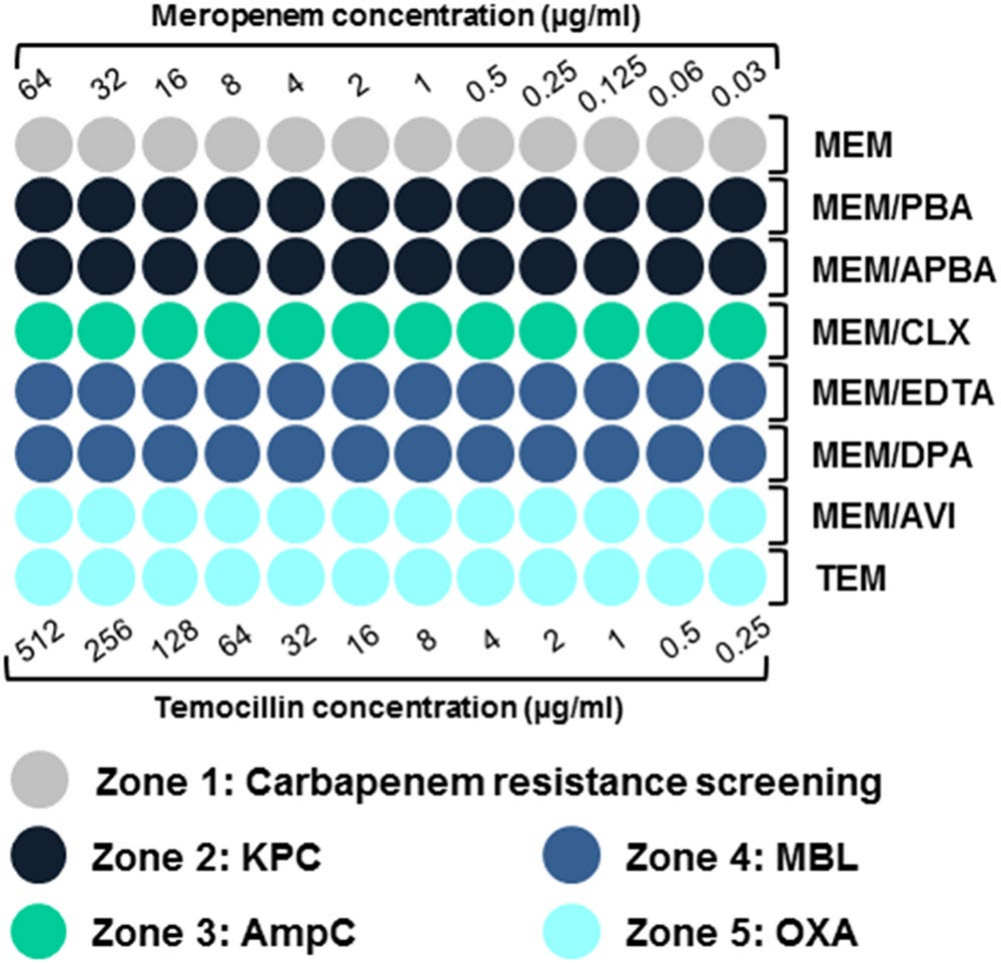
Layout of the DOT-MGA screening panel. The mass spectrometric assessment of bacterial growth on each spot allows the MIC determination for each row. Significant MIC decrease (8-fold or more) in zones 2–5 in relation to zone 1 indicates presence of a certain carbapenemase. Temocillin MIC > 128 µg/ml (last row) is compatible with OXA production MEM: meropenem; PBA: phenylboronic acid; APBA: aminophenylboronic acid; CLX: cloxacillin; EDTA: ethylendiamintetraacetic acid; AVI: avibactam; TEM: temocillin.

The DOT-MGA screening panel was developed as a one-step method for detection of carbapenem non-susceptibility, AmpC production and carbapenemase class differentiation. It was performed on a 96-well MALDI-TOF target (Bruker Daltonik, Bremen, Germany), following the layout depicted in Fig. 1. A total of 6 µl containing a suspension of the tested strain and meropenem in serial two-fold dilutions was pipetted onto each of the target’s spots, with each row containing antibiotic concentrations ranging from 0.03 to 64 µg/ml. In order to differentiate between carbapenemase classes, specific inhibitors were added in several rows. After incubating the target and removing the broth, the meropenem minimum inhibitory concentration (MIC) of each row could be determined by assessing the bacterial growth on the spots through a mass spectrometric analysis. High temocillin resistance, common in OXA-producing isolates, was determined by this same principle in the panel’s last row (concentrations 0.25 to 512 µg/ml). The meropenem concentration present in the first spot (in ascending order) of a given row that showed no bacterial growth was considered the MIC. A significant decrease of the meropenem MIC (8-fold or more) in rows with added carbapenemase inhibitors was interpreted as an indicator of a synergistic effect, allowing for the differential identification of carbapenemase classes.

The validation of the method was performed on seven reference strains recommended by EUCAST for detection of carbapenemases^24^ (Table 1), as well as on 20 meropenem non-susceptible *Enterobacterales* strains isolated from clinical samples (Table S1). The method was carried out with two different incubation times, namely 3 and 4 hours, observing a significantly higher accuracy at the second time point. The results of DOT-MGA, as well as of BMD and CDT, were evaluated considering the PCR as an imperfect standard (accepted method of comparison which may be imprecise to some extent)^25^, calculating the positive percent agreement (PPA) and negative percent agreement (NPA) for each method (Table 2):

**Table 1:** Detection performance of the DOT-MGA screening panel on reference strains recommended by EUCAST.

Strain	Foreknown resistance mechanism, confirmed by PCR	DOT-MGA screening panel result (4 h)
*K. pneumoniae* NCTC 13438	KPC	KPC
*E. cloacae* CCUG 56927	AmpC + porin loss	AmpC
*K. pneumoniae* NCTC 13440	MBL (VIM)	MBL
*K. pneumoniae* NCTC 13443	MBL (NDM-1)	MBL
*E. coli* 13476	MBL (IMP)	MBL
*K. pneumoniae* 13442	OXA-48	OXA-48
*E. coli* ATCC 25922	None	None

**Table 2:** Detection performance of DOT-MGA, BMD and CDT on clinical isolates compared to PCR.

Resistance mechanism	Detection method (incubation time)							
	DOT-MGA (4 h)		DOT-MGA (3 h)		BMD (18 h)		CDT (18 h)	
	PPA	NPA	PPA	NPA	PPA	NPA	PPA	NPA
Carbapenem resistance	100%	100%	70%	100%	80%	80%	90%	10%
KPC	— *	100%	— *	95%	— *	90%	— *	65%
AmpC	33.3%	88.2%	33.3%	94.1%	33.3%	82.4%	33.3%	82.4%
MBL	100%	100%	50%	100%	75%	100%	75%	100%
OXA	100%	100%	71.4%	100%	85.7%	84.6%	100%	53.9%

^*^No PPA available as KPC was not detected in any of the tested clinical isolates.

### DOT-MGA accurately detected carbapenem non-susceptibility

After 4 hours of incubation, DOT-MGA was able to detect carbapenemase activity in the 6 carbapenemase-producing reference strains as well as in 10 of 10 isolates with carbapenemase production confirmed by PCR. PPA and NPA values of 100% were reached, as opposed to those shown by BMD (80%/80%) and CDT (90%/10%).

### DOT-MGA correctly identified KPC, MBL and OXA carbapenemases

The foreknown mechanisms of resistance present in all control strains (KPC; MBL; OXA and AmpC) were correctly identified by DOT-MGA after 4 hours of incubation (Table 1).

The identification of carbapenemase classes among the clinical isolates tested was also most accurate after 4 hours of on-target incubation. Aside from one KPC-positive control strain, KPC production was not detected by DOT-MGA in clinical isolates, in accordance with the PCR results (Table S1); this translated into an NPA of 100% (BMD: 90%; CDT: 65%). DOT-MGA displayed a PPA of 100% for the detection of MBL, in comparison to 75% reached by BMD and CDT. All three methods correctly identified MBL-negative isolates with an NPA of 100%. OXA production was detected by DOT-MGA with PPA and NPA values of 100%. BDM and CDT yielded several false positive results (2 and 6, respectively), leading to a significantly lower NPA. Strains were also analysed for AmpC production as possible additional mechanism of resistance. Here, all three methods performed similarly, identifying two (DOT-MGA) to three (BMD, CDT) isolates as AmpC-positive while they were negative by PCR.

## Discussion

Carbapenem resistance among Gram-negative microorganisms is a global threat with increasing significance for human and veterinary public-health and with growing environmental concerns, e.g. as hospital wastewater contaminants^26–28^. An innovative phenotypic method is presented that offers concrete information about the carbapenem non-susceptibility *Enterobacterales* isolates. The DOT-MGA screening panel has been designed in an easy-to-perform format that allows testing for several mechanisms of carbapenem non-susceptibility in a single step.

Our approach detected type-specific carbapenemase production with a performance equivalent to that of the PCR and a higher accuracy than that of the other phenotypic methods evaluated, identifying specific carbapenemase types.

KPC production was successfully detected in one control strain, with no false positive results among the clinical strains, all of which tested negative for KPC by PCR. MBL was successfully detected in all 4 clinical isolates also identified by PCR, despite the additional production of AmpC in two of them. With high PPA and NPA values of 100%, the test performed satisfactorily in terms of OXA detection, a worldwide spread carbapenemase class of special relevance in Germany^12,29^ which often poses a diagnostic challenge^30,31^. The combination of two detection principles, synergy of meropenem with avibactam and high-level temocillin resistance, allowed overcoming the possible masking effect of MBL in an isolate producing both carbapenemases.

In addition to the identification of carbapenemases, the detection of AmpC production was included in the screening panel as a complementary feature, due to the decreased susceptibility to carbapenems that these enzymes may cause^32–34^. DOT-MGA coincided with the PCR in one AmpC-positive isolate. However, two further isolates negative for AmpC in the PCR showed positive DOT-MGA results, as they proved susceptible to the combination meropenem/cloxacillin. In both cases, BMD and CDT also delivered AmpC-positive results. An explanation for this, besides possible false positive results, is the presence of AmpC genes in the isolates that were not detected by the PCR microarray.

The proposed assay offers a one-step method that allows for (i) determination of carbapenem non-susceptibility; (ii) MIC quantification; and (iii) specific carbapenemase detection. Being a phenotypic approach, it allows the detection of unknown or uncommon carbapenemases encoded by uncommon or emerging genes not identified by routine DNA-based methods. While designed as proof-of-principle study delivering primary data on a new technique, a limitation is the small number of isolates tested and the low carbapenemase diversity. This is in part a result of the strictly consecutive collection of clinical strains at the routine laboratory, thus reflecting the German epidemiological situation^12^. Further evaluation on clinical isolates producing diverse carbapenemases, especially KPC, would be useful in order to complement the validation process carried out in the present study. Also, customised assay equipment and adapted analysis software may further enhance the standardisation, manageability and performance of this approach.

Overall, the screening panel identified the main carbapenemase classes with a higher accuracy than other phenotypic methods routinely used, yielding results comparable to those of the genotypic reference method (PCR). This assay provides detailed and reliable type-specific carbapenemase detection after 4 hours, constituting a valuable tool for the acceleration of microbiological diagnostic procedures. The method can be easily implemented in laboratories working with MALDI-TOF MS, since it does not require additional instrumentation and the testing procedure is similar to that of the regular MALDI-TOF MS identification. In future phases of development, alternative preparation of the necessary stock solutions (i.e. pre-coated targets) could facilitate the automation of the method, further reducing the turnaround time. Being performed as a routine technique, the DOT-MGA screening panel would expedite the decision-making process in the healthcare setting, contributing to a more appropriate approach to carbapenem-resistant microorganisms at both the clinical and epidemiological level. This would translate into better clinical outcomes, a more rational use of carbapenems and faster implementation of infection control measures.

## Methods

### Bacterial strains

For the development of the panel, preliminary experiments were performed on 6 carbapenem-resistant strains recommended by EUCAST for the detection of carbapenemase production^24^ and one non-carbapenemase-producing strain used as a negative control (Table 1).

In a second phase, the panel was challenged with a total of 20 *Enterobacterales* strains (Table S1) consecutively isolated from clinical samples processed at the Institute of Medical Microbiology of the University Hospital Münster, Germany. These isolates were routinely identified by MALDI-TOF MS and displayed resistance against meropenem^35^ as determined by the Vitek 2^®^ card AST-N214 (bioMérieux, Marcy l’Etoile, France).

Bacterial suspensions were prepared and adjusted to a density of 0.5 McFarland employing a nephelometer (Densimat, bioMérieux, Marcy l’Etoile, France). Subsequently, a dilution 1:100 was made with cation-adjusted Mueller-Hinton broth (CA-MHB).

### Antimicrobial substances

Given its high sensitivity and specificity^36,37^, meropenem (TCI Deutschland GmbH, Eschborn, Germany) was used as indicator substance to screen for carbapenem non-susceptibility. The detection of each carbapenemase class relied on the synergy of meropenem with the following specific carbapenemase inhibitors: phenylboronic acid (PBA, TCI), aminophenylboronic acid (APBA, TCI), cloxacillin (CLX, TCI), dipicolinic acid (DPA, TCI), ethylenediaminetetraacetic acid (EDTA, TCI) and avibactam (Advanced ChemBlocks Inc., Burlingame, CA, USA). Temocillin (Eumedica Pharmaceuticals, Basel, Switzerland) was used as an indicator for OXA production. Stock solutions of each substance were prepared following the guidelines of the Clinical and Laboratory Standards Institute (CLSI)^38^ using deionised distilled water. Due to the physicochemical properties of PBA and APBA, a dilution in dimethyl sulfoxide (AppliChem, Darmstadt, Germany) and deionised distilled water (mixed in a 1:1 ratio) was necessary. The quality of the meropenem stock solution was tested according to the specifications of CLSI^38^ and EUCAST^39^, verifying the MIC of *E. coli* ATCC 25922. Seeing as how no quality standards have been established for the rest of the substances employed, the working concentrations were standardised in triplicate.

### Screening panel

The panel was designed to comprise several zones distributed over a 96-spot format as shown in Fig. 1. Zone 1, a two-fold dilution series of meropenem constituted the primary carbapenem non-susceptibility screening. Zones 2, 3 and 4 contained meropenem in combination with specific inhibitors, allowing for the detection of three carbapenemase classes: KPC (PBA, APBA), AmpC (CLX), MBL (EDTA, DPA). Zone 5 included two different methods of OXA detection based on the synergy between meropenem and avibactam^40^, as well as on the high-level temocillin resistance (>128 µg/ml) frequently observed in OXA-producing strains^24,41^. The appropriate concentrations of the different inhibitors were defined in internal standardisation tests partly based on previous reports^42,43^.

DOT-MGA was performed as previously described^23^. Microdroplets (total volume 6 µl) containing 3 µl of bacterial suspension (final inoculum approximately 5 × 10^5^ CFU/ml) and 3 µl of the different antimicrobial solutions (meropenem, meropenem/inhibitor and temocillin) were distributed on the spots of an MBT Biotarget 96 (Bruker Daltonik, Bremen, Germany). A second target was used for the sterility and growth controls.

Targets were incubated for 3 or 4 hours at 35 ± 1 °C. In order to avoid the evaporation of the microdroplets, targets were kept in plastic transport boxes (Bruker Daltonik) containing water during the incubation. Afterwards, broth was carefully removed from the spots employing filter paper (size 37 × 100 mm, GE Healthcare GmbH, Freiburg, Germany), avoiding cross contamination of the microdroplets. The spots were then overlaid with 1 µl of α-cyano-4-hydroxycinnamic acid matrix including internal standard (MBT MASTeR prototype kit, Bruker Daltonik) and MALDI-TOF MS spectra were acquired on a microflex smart instrument (Bruker Daltonik). The assay was performed in triplicate. The median of the values obtained in each measurement was used for further interpretation.

The final carbapenemase detection was based on the comparison and combined analysis of the minimum inhibitory concentration (MIC) detected for each substance or combination of substances tested on the panel.

By processing the spectra obtained from the MALDI-TOF MS readings with the MALDI Biotyper Software 3.1 (Bruker Daltonik), bacterial growth was identified on those spots where the tested strain was not inhibited by the antimicrobial substances used. In contrast, spots on which antibiotics achieved an inhibitory effect (i.e. because of higher concentrations or synergistic combination), no growth was identified. Based on the degree of growth detection, a score was automatically assigned to each spot, with a score ≥2.0 being considered indicative of bacterial growth. Following the principle of the classic broth microdilution method (BMD), the MIC of the antibiotics in presence or absence of inhibitors was defined as the lowest concentration at which no bacterial growth could be detected by MALDI-TOF MS, corresponding to a detection score <2.0.

The meropenem MIC in zone 1 (Fig. 1) was interpreted according to the EUCAST breakpoints and screening cut-off values defined in the guidelines for detection of resistance mechanisms^24,35^ in order to classify isolates in susceptible (MIC ≤ 0.125), putative carbapenemase-producers (0.125 > MIC ≤ 2), intermediate (2 > MIC ≤ 8) or resistant (MIC > 8) towards meropenem.

The identification of specific carbapenemase classes was based on the synergy between meropenem and the respective carbapenemase inhibitor, indicated by an 8-fold decrease (or more) of the initial meropenem MIC. The carbapenemase identification employing two inhibitors (KPC, MBL) was considered correct when synergy was observed in both cases. A further parameter was considered for the detection of OXA production, namely a high-level temocillin resistance (>128 µg/ml). MIC values were processed and interpreted using a computer-based algorithm in order to obtain a final result.

### Complementary methods of detection of carbapenem non-susceptibility

BMD was performed on 96-well microtiter plates following the specifications of CLSI^38^ and the International Organization for Standardization^44^, following the same layout of the DOT-MGA panel. Stock solutions containing meropenem and carbapenemase inhibitors where prepared as described above. The concentrations of the different inhibitors were adapted for the recommended incubation time (18 ± 2 h), longer than that required for DOT-MGA. Bacterial suspension (30 µl) and antibiotic solutions (30 µl) were distributed in the wells and sterility and growth controls were carried out on a second plate. After incubation for 18 ± 2 h at 35 ± 1 °C, turbidity was assessed. MIC was defined as the lowest concentration showing no turbidity. Synergy between meropenem and carbapenemase inhibitors was defined by an 8-fold decrease (or more) of the initial meropenem MIC, indicating the production of a specific carbapenemase class. The method was performed in triplicate and median values were determined.

Strains were also tested with a phenotypic combination disk test (Mast Diagnostica GmbH, Reinfeld, Germany), the carbapenemase-set D70C (meropenem only and combined with MBL, KPC and AmpC inhibitors), as well as disks containing 30 µg of temocillin. Tests were performed and results interpreted according to the manufacturer’s instructions. In short, bacterial suspensions of the analysed strains (McFarland 0.5) were spread on Mueller-Hinton agar plates (BD, Heidelberg, Germany) and disks were placed onto the agar leaving enough space for inhibition zones to be seen correctly. After incubating at 35–37 °C for 18 hours, the inhibition zones were measured and interpreted accordingly.

All clinical strains tested were genotypically characterised using the PCR microarray Check-MDR CT103 XL (Check-Points, Wageningen, The Netherlands). DNA was isolated and the array was performed according to the manufacturer’s instructions. Targeted genes are summarised in Table S2.

### Statistical analysis

The PCR was considered an imperfect standard^25^ for the evaluation of the DOT-MGA. BMD and CDT were compared with PCR as well. Positive and negative percent agreements (PPA and NPA, respectively) of all three methods were calculated according to the statistical guidance of the Food and Drug Administration^45^.

The data were presented in part on ECCMID 2018, Madrid, 21–24 April (#5622) and IDWeek 2018, San Francisco, 03–07 October (#2066).

## Supplementary information

Supplementary Data - Tables S1 and S2.

## Data Availability

All data generated or analysed during this study are included in this published article (and its Supplementary Information Files).
