# Supplementary material for: Development of a MALDI-TOF MS-based screening panel for accelerated differential detection of carbapenemases in Enterobacterales using the direct-on-target microdroplet growth assay
Source: Sci Rep. 2020 Mar 19;10:4988. doi: 10.1038/s41598-020-61890-7 (PMC7081182; doi:10.1038/s41598-020-61890-7)
Supplement: Supplementary file 1 — Supplementary Data - Tables S1 and S2. [file 41598_2020_61890_MOESM1_ESM.pdf]

**Development of a MALDI-TOF MS-based screening panel for accelerated differential detection of carbapenemases in *Enterobacterales* using the direct-on-target microdroplet growth assay**

Carlos L. Correa-Martínez, Evgeny A. Idelevich, Katrin Sparbier, Thorsten Kuczius, Markus Kostrzewa, Karsten Becker

**Table S1.** Resistance mechanisms detected by BMD, CDT, DOT-MGA (3 and 4 hours of incubation) and PCR in 20 clinical *Enterobacterales* isolates.

| Isolate | BMD | CDT | DOT-MGA | | PCR | |
|---|---|---|---|---|---|---|
| | | | 3 h | 4 h | Result | Detected gene(s) |
| *K. pneumoniae* | OXA | OXA | KPC | OXA | OXA | OXA-48/ CTX-M-9 |
| *K. pneumoniae* | - | KPC/OXA | - | - | - * | CTX-M-15 |
| *E. aerogenes* | KPC/AmpC | KPC/AmpC/ OXA | - | - | - | - |
| *K. pneumoniae* | MBLOXA | MBL/OXA | MBL | MBL | MBL* | NDM/ CTX-M-15 |
| *K. pneumoniae* | - | OXA | - | - | - * | CTX-M-15 |
| *K. pneumoniae* | - | OXA | OXA | OXA | OXA | OXA-48 |
| *K. pneumoniae* | OXA | OXA | OXA | OXA | OXA* | OXA-48/ CTX-M-9 |
| *P. mirabilis* | - | - | - | - | - | - |
| *K. pneumoniae* | - | KPC | - | - | - * | CTX-M-15 |
| *K. oxytoca* | MBL/OXA | - | - | MBL | MBL | VIM |
| *E. cloacae* | AmpC | KPC/AmpC | AmpC | AmpC | AmpC* | ACT/MIR/ CTX-M-9 |
| *E. aerogenes* | KPC/AmpC | KPC | AmpC | AmpC | - | - |
| *E. coli* | MBL/OXA | MBL/OXA | - | MBL/OX A | MBL/OXA/ AmpC | OXA-48/NDM/ CMY-2 |
| *K. pneumoniae* | OXA | OXA | - | OXA | OXA* | OXA-48/ CTX-M-15 |
| *E. cloacae* | AmpC | KPC/AmpC/ OXA | - | AmpC | - | - |
| *K. pneumoniae* | - | OXA | - | - | - * | CTX-M-15 |
| *K. pneumoniae* | OXA | OXA | OXA | OXA | OXA* | OXA-48/ CTX-M-15 |
| *K. pneumoniae* | OXA | OXA | OXA | OXA | OXA* | OXA-48/ CTX-M-15 |
| *K. pneumoniae* | - | - | MBL | MBL | AmpC/MBL* | NDM/CMY-2 |
| *P. mirabilis* | - | - | - | - | - | - |

- : Negative result.

* : Additional detection of ESBL production.

**Table S2.** Genes detected by the PCR microarray.

| Carbapenemases | | ESBL | | | | Minor ESBL | AmpC |
|---|---|---|---|---|---|---|---|
| GES* | OXA-24 | CTX-M-1 group | CTX-M-9 group | TEM wt | SHV wt | BEL | ACC |
| GIM | OXA-48 | CTX-M-1 subgroup | CTX-M-15 subgroup | TEM 104K | SHV 238A | GES** | ACT/MIR |
| IMP | OXA-58 | CTX-M-2 group | CTX-M-25 group | TEM 164C | SHV 238S | PER | CMY-1/ MOX |
| KPC | VIM | CTX-M-3 subgroup | CTX-M-32 subgroup | TEM 164H | SHV 240K | VEB | CMY-2 |
| NDM | SPM | CTX-M-8 group | | TEM 164S | | | DHA |
| OXA-23 | | | | TEM 238S | | | FOX |

*GES, enzymes with carbapenemase activity (2, 4-6, 13-15, 17, 18, 20, 21);

**GES, enzymes with ESBL activity (1, 3, 7-12, 16, 19, 22).
